# Functional Expression and Characterization of a Panel of Cobalt and Iron-Dependent Nitrile Hydratases

**DOI:** 10.3390/molecules25112521

**Published:** 2020-05-28

**Authors:** Birgit Grill, Maximilian Glänzer, Helmut Schwab, Kerstin Steiner, Daniel Pienaar, Dean Brady, Kai Donsbach, Margit Winkler

**Affiliations:** 1 Austrian Center of Industrial Biotechnology GmbH, 8010 Graz, Austria; birgit.grill@tugraz.at (B.G.); maximilian.glaenzer@student.tugraz.at (M.G.); helmutschwab@acib.at (H.S.); kerstin.steiner@acib.at (K.S.); 2 Molecular Science Institute, School of Chemistry, University of the Witwatersrand, P.O. Wits 2050, Johannesburg, South Africa; daniel.pienaar@wits.ac.za (D.P.); Dean.Brady@wits.ac.za (D.B.); 3 PharmaZell, 83064 Raubling, Germany; kai.donsbach@pharmazell.com; 4 Institute of Molecular Biotechnology, Graz University of Technology, NAWI Graz, 8010 Graz, Austria

**Keywords:** nitrile hydratase (NHase), nitrile, amide, metalloprotein, non-heme iron, non-corrinoid cobalt, biocatalysis, active pharmaceutical ingredient.

## Abstract

Nitrile hydratases (NHase) catalyze the hydration of nitriles to the corresponding amides. We report on the heterologous expression of various nitrile hydratases. Some of these enzymes have been investigated by others and us before, but sixteen target proteins represent novel sequences. Of 21 target sequences, 4 iron and 16 cobalt containing proteins were functionally expressed from *Escherichia coli* BL21 (DE3) Gold. Cell free extracts were used for activity profiling and basic characterization of the NHases using the typical NHase substrate methacrylonitrile. Co-type NHases are more tolerant to high pH than Fe-type NHases. A screening for activity on three structurally diverse nitriles was carried out. Two novel Co-dependent NHases from *Afipia broomeae* and *Roseobacter sp.* and a new Fe-type NHase from *Gordonia hydrophobica* were very well expressed and hydrated methacrylonitrile, pyrazine-carbonitrile, and 3-amino-3-(*p*-toluoyl)propanenitrile. The Co-dependent NHases from *Caballeronia jiangsuensis* and *Microvirga lotononidis*, as well as two Fe-dependent NHases from *Pseudomonades*, were—in addition—able to produce the amide from cinnamonitrile. Summarizing, seven so far uncharacterized NHases are described to be promising biocatalysts.

## 1. Introduction

**Figure 1:**
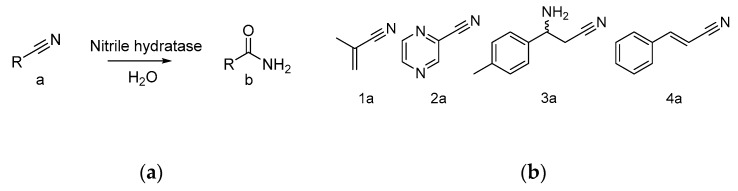
(**a**) Reaction scheme; (**b**) substrates investigated in this study.

Nitrile hydratases (NHase; EC 4.2.1.84) catalyze the hydration of nitriles to the corresponding amides (Figure 1a) [1]. Due to the broad substrate acceptance of NHases, numerous amides are accessible from their respective nitrile precursors [2]. The first example of an industrial bioconversion process for the manufacturing of the commodity chemical acrylamide involved NHase in a microbial host [3]. Nicotinamide and 5-cyanoveleramide are also products made on a commercial production scale with NHase biocatalysts [4]. Another important field of application of these enzymes is the bioremediation of contaminating nitriles [5]. A review from earlier this year summarizes NHase research from recent years, including the natural distribution, gene types, post-translational modifications, expression, proposed catalytic mechanism, biochemical properties, and potential applications of NHases [4]. Briefly, two types of NHases can be distinguished, the non-corrin cobalt-containing and non-heme iron-containing NHases. Both types are composed of an α- and a β-subunit and functional heterologous expression depends on the action of an accessory protein [6,7]. Successful heterologous expression has been described for the NHases from *Klebsiella oxytoca* [8], *Bacillus sp. RAPc* [9], and the thermostable NHases from *Pseudonocardia thermophila* [10], *Pseudomonas putida* [6], and *Aurantimonas manganoxydans* [11]. However, limited acceptability of substrates, low expression levels, and instability under process relevant conditions may still hamper practical applications of NHase biocatalysts.

Herein we explored the sequence space, cloned, and expressed a few known and sixteen so far uncharacterized proteins with sequence similarity to nitrile hydratases. The investigated panel consists of seven Fe-type NHases and 16 Co-type NHases. Functional expression and basic characterization regarding substrate scope and selected biochemical characteristics are reported.

## 2. Results and Discussion

### 2.1. Identification and Production of Nitrile Hydratases

**Figure 2:**
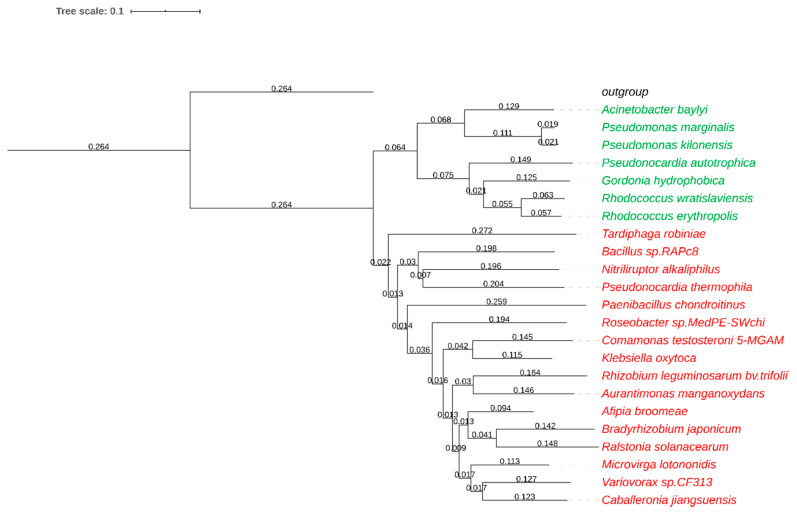
Phylogenetic relationship of nitrile hydratase (NHase) α-subunits based on a protein distance analysis with T-COFFEE (Version_11.00.d625267) and visualization with iTOL (Version 5.5.1) [12]. *Rhodococcus erythropolis* amidase (Uniprot: P22984.2) served as the outgroup. Iron-type NHases are displayed in green, cobalt-type NHases in red.

With the aim to identify new nitrile hydratases, we looked for described NHase enzymes in the literature and in databases (NCBI, pdb). Reported heterologous expression in *Escherichia coli* and high thermostability were favored properties of literature-known NHases. In addition, the sequences of the well-studied iron-type *Re*NHase and cobalt-type *Br*NHase were used as templates for a blastp search against the non-redundant GenBank protein sequences. Several sequences, assigned as (putative) nitrile hydratases, were subjected to computational analysis regarding presence of a respective (putative) accessory protein, lack of transmembrane regions and PEST motifs (proline, glutamate, serine, and threonine rich motives which may cause rapid protein degradation), and the optimal growth temperature of their source organism, whereby enzymes of psychrophilic organisms were rejected. We selected diverse sequences as summarized in Figure 2. The NHase genes were cloned into the pMS470 vector and expressed under the control of the tac promoter in *E. coli* in the presence of their putative accessory proteins. The active site metal was supplemented at the time of induction of gene expression. The expression temperature was 20 °C to facilitate protein folding and metal incorporation.

High expression levels were achieved for *Microvirga lotononidis* NHase (*Ml*NHase), *Paenibacillus chondroitinus* NHase (*Pc*NHase), *Rhodococcus erythropolis* NHase (*Re*NHase), *Caballeronia jiangsuensis* NHase (*Cj*NHase), *Gordonia hydrophobica* NHase (*Gh*NHase), and *Bacillus sp.* RAPc8 NHase (*Br*NHase) in the presence of 0.1 mM CoCl_2_ or 1 mM FeSO_4_ [9] (Figures S1 and S2). *Bradyrhizobium japonicum* NHase (*Bj*NHase), *Aurantimonas manganoxydans* NHase (*Am*NHase) [13], and *Pseudonocardia thermophila* NHase (*Pt*NHase) [14] were less well expressed. The *Klebsiella oxytoca* NHase (*Ko*NHase) [8] showed imbalanced overexpression with significant amounts of the β-subunit but almost no α-subunit. *Nitriliruptor alkaliphilus* NHase (*Na*NHase) [15] predominantly formed inclusion bodies and was detected in the insoluble fraction. A putative NHase from *Rhizobium leguminosarum bv. trifolii* (*Rl*NHase) was found in the soluble fraction in minor amounts only when 1 mM of CoCl_2_ was supplemented (Figure S5). Overexpression was also achieved for *Afipia broomeae* NHase (*Ab*NHase), *Comamonas testosteroni* 5-MGAM NHase (*Ct*NHase) [16], *Roseobacter sp.* MedPE-SWchi NHase (*Rm*NHase), and Variovorax sp. CF313 (VvNHase) (Figure S3). The putative NHases from *Ralstonia solanacearum* (*Rs*NHase) and from *Tardiphaga robiniae* (*Tr*NHase) showed higher expression of the α- subunit as compared to the β-subunit. The iron-type NHase from *Pseudomonas kilonensis* (*Pk*NHase) was well expressed whereas the NHase from *Acinetobacter baylyi* (*Ac*NHase) was found neither in the soluble nor in the insoluble fraction. *Pseudomonas marginalis* NHase (*Pm*NHase) showed imbalanced expression with higher amount of the β-subunit (Figure S3).

### 2.2. Activity for Methacrylonitrile and Characterization of pH Optimum and Stability

Cell free extracts (CFE) were subsequently used to investigate whether the NHases were capable to hydrolyze a typical NHase substrate, methacrylonitrile (MAN, **1a**) [17]. This conversion can conveniently be monitored in a plate reader, allowing efficient investigation of the effect of different reaction parameters, and was therefore applied where appropriate. Table 1 shows that expression levels do not necessarily correlate with activities. This fact indicates that some NHases may be particularly active on small aliphatic substrates like MAN (e.g., *Re*NHase) whereas others might prefer substrates of different chemical structure (e.g., *Gh*NHase, which is produced in high amounts but does not hydrate MAN very well, see Table 1) Similarly, Co-dependent *Ko*NHase readily produced methacryloamide, whereas *Ml*NHase, although better expressed, was not as efficient with MAN as the substrate. Hence, we expected diverging substrate specificities of the NHase panel. Outstanding activities for the substrate MAN were obtained with the two iron-dependent NHases from *R. erythropolis* and *P. kilonensis* and the two cobalt-dependent NHases from *K. oxytoca* and *C. testosteroni*. The activities in relation to the amount of NHase, which was estimated from gel pictures, revealed also Co-type *Na*NHase, and Fe-type *Gh*NHase and *Pm*NHase as promising candidates.

**Figure 3:**
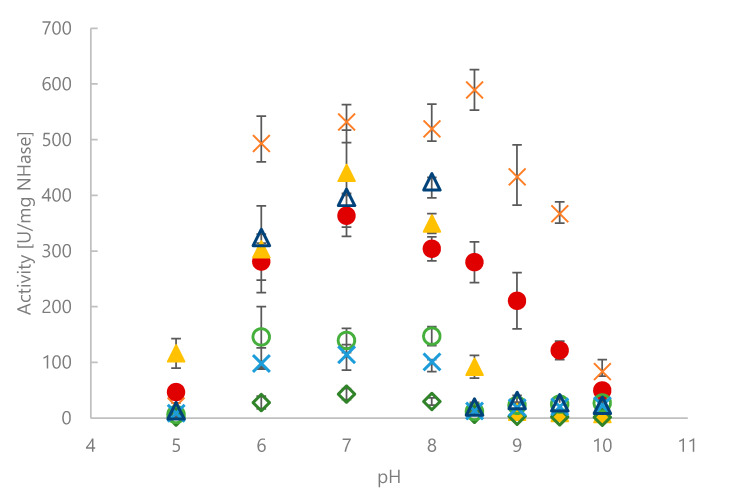
Activities of NHase CFEs for MAN hydrolysis at different pH values and 25 °C. Citrate-phosphate buffer pH 5–6, sodium phosphate buffer pH 7–8, Tris-HCl buffer pH 8.5, carbonate buffer pH 9–10. *Ct*NHase: filled dark red circles. *Ko*NHase: filled orange squares. *Na*NHase: filled yellow triangles. *Gh*NHase: green rhombs. *Pk*NHase: light green circles. *Pm*NHase: blue crosses. *Re*NHase: dark blue triangles.

The spectrophotometric MAN assay was used to determine biochemical characteristics of seven selected NHases. The effect of pH is summarized in Figure 3. Fe-dependent NHases showed good activities between pH 6.0 and 8.0, but already pH 8.5 fully abolished activity. Co-dependent NHases tolerated a broader pH range, although activities decreased significantly above pH 8.5.

**Figure 4:**
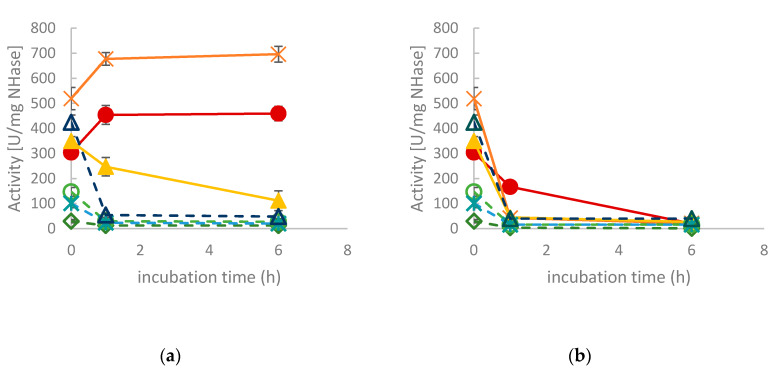
Residual activities of NHase CFEs for MAN hydrolysis at 25 °C. (**a**) After incubation at 37 °C in buffer at pH 8.0; (**b**) after incubation at 50 °C at pH 8.0. *Ct*NHase: filled dark red circles. *Ko*NHase: filled orange squares. *Na*NHase: filled yellow triangles. *Gh*NHase: green rhombs, dashed line. *Pk*NHase: light green circles, dashed line. *Pm*NHase: blue crosses, dashed line. *Re*NHase: dark blue triangles, dashed line.

In view of future applications, the operational stability of the enzymes is an important parameter. Therefore, the NHase containing CFEs were incubated at 37 °C and 50 °C, respectively. Residual activity was determined after 1 and 6 h, respectively. As shown in Figure 4a, *Ct*NHase and *Ko*NHase retained full activity when incubated at 37 °C, whereas all other NHases were deactivated to some degree upon incubation (Figure 4a). The effect was even more pronounced upon treatment at 50 °C. This temperature was only tolerated by *Ct*NHase to some extent (Figure 4b).

To investigate whether activity loss was caused by the denaturation of the NHases, the proteins in form of CFEs were treated at 37 °C and 50 °C for 16 h, respectively. After centrifugation, the soluble fraction was analyzed by SDS-PAGE. *Pm*NHase and *Pk*NHase had almost completely precipitated at both temperatures (Figure S4). The soluble amounts of *Ct*NHase, *Ko*NHase, *Gh*NHase and *Na*NHase decreased upon incubation, especially at 50 °C. Remarkably, the amount of soluble *Re*NHase remained constant, which indicates that aggregation was not the reason for activity loss of this particular NHase.

### 2.3. Exploration of Substrate Scope

**Figure 5:**
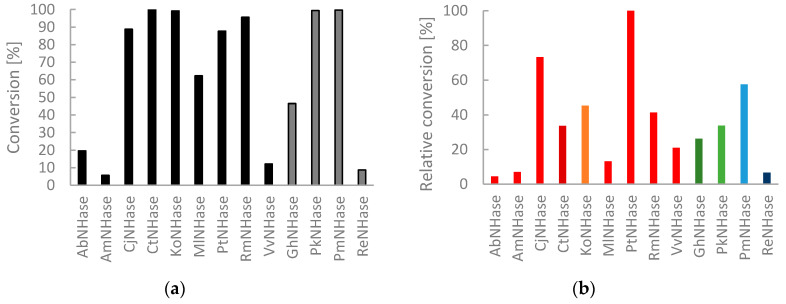
(**a**) Amide formation from 5 mM **2a** with NHase CFEs (1:200 dilution) in 100 mM KH_2_PO_4_ buffer at pH 7.2 and 25 °C. Reaction time 5 min. *Na*NHase was not included. Calculation was based on area normalization. All omitted NHases were inactive for **2a** hydration under these conditions. Black: Co and Grey Fe-dependent NHases; (**b**) Relative activities of NHases. Red tones: Co- and Green/Blue Fe-dependent NHases.

To evaluate the potential of the NHase panel further, we selected structurally diverse nitriles and used them as substrates in biotransformations with cell free extracts. The conversion of pyrazine-2-carbonitrile (**2a**) gives pyrazine-2-carboxamide, which is known to be an antitubercular agent [18]. All NHases were included in this screening for activity. Conversions were determined by high performance liquid chromatography (HPLC) with mass selective detection (MS). Initial experiments gave full conversion of **2a** with all NHase CFEs. To identify differences, we diluted the protein preparations and shortened reaction times. Indeed, a ranking of biocatalysts was then feasible and four NHase CFEs gave >99% conversion (C*t*NHase, *Ko*NHase, *Pk*NHase and *Pm*NHase) (Figure 5a). Considering the varying amounts of NHase in the respective CFE, Figure 5b shows a comparison with normalized amounts of NHase. Therefore, expression levels were estimated from gel pictures. For **2a**, the NHase from *Pseudonocardia thermophila* is the most active NHase of the group of Co-dependent enzymes and *Pseudomonas marginalis* the most efficient amongst Fe-dependent NHases.

**Figure 6:**
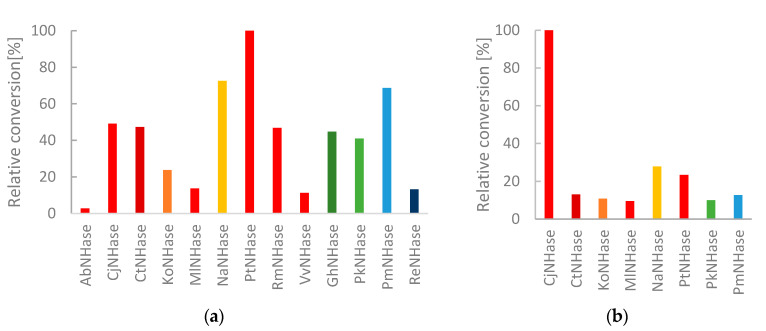
Amide formation from 5 mM substrate. Reaction time 5 min. Red tones: Co- and Green/Blue Fe-dependent NHases. (**a**) **3a** with NHase CFEs (1:200 dilution) in 100 mM KH_2_PO_4_ buffer at pH 7.2 and 25 °C. All missing NHases were inactive for **3a** hydration under these conditions. (**b**) **4a** with NHase CFEs (1:100 dilution) in 273 mM Na_2_HPO_4_/72 mM NaH_2_PO_4_ at pH 7.5. All omitted NHases were inactive for **4a** hydration under these conditions.

3-Amino-3-(p-toluoyl)propanenitrile (**3a**) belongs to the group of β-amino nitriles whose corresponding amides are of great pharmaceutical importance as they serve as building blocks for the synthesis of both biologically active peptides and small molecule pharmaceuticals. Both **3a** and **2a** have previously been successfully subjected to enzymatic hydration using a *R. rhodochrous* nitrile hydratase [19,20]. β-Amino nitriles can also be transformed to amides and carboxylic acids with protective groups on the β-amino group using the native NHase and amidase expressing host organisms [21,22,23]. Normalized on NHase content, *Pseudonocardia thermophila* and *Pseudomonas marginalis* showed high potential for the conversion of this primary amine containing substrate 3-amino-3-(p-tolyl)propanenitrile (**3a**) (Figure 6a). When equal amounts of CFEs were used, **3a** was fully converted to the respective amide by *Ct*NHase, due to its exceptional expression level (data not shown).

To extend the structural scope of substrates, we furthermore looked into the capability of the NHases to convert the unsaturated cinnamic acid nitrile (**4a**) [24]. Of all NHases, *Cj*NHase showed clearly outstanding activity for compound **4a** (Figure 6b). For **3a**, *Cj*NHase showed only little activity (Figure 6a) and for **2a**, the enzyme was good, but not excellent (Figure 5b). A much less pronounced trend was observed for compound **3a**, which was hydrated to a high degree by Co-dependent *Ct*NHase, *Rm*NHase and *Pt*NHase, but also by the Fe-dependent *Pm*NHase. The heterocyclic pyrazine nitrile **2a**, was converted very efficiently by seven NHases of both types. A concentration of 5 mM pyrazine-2-carbonitrile (**2a**) could, for example, be fully hydrated by only 9 µg/mL *Ko*NHase in the reaction mixture within less than 5 min. Similarly, 12 µg/mL *Ct*NHase produced 5 mM of **3b** and 10 µg/mL *Cj*NHase 5 mM of **4b**.

The screening results with methacrylonitrile (**1a**) (Table 1) agreed with these end-point measurements in terms of unsuitable NHases (those with low activities for MAN were generally also poor for other substrates). The MAN assay is a suitable tool to quickly reveal highly active NHase preparations capable of converting other nitriles efficiently as well, although this assay may overestimate the potential of NHases with a particular preference for small, aliphatic nitriles.

In conclusion, cobalt-dependent nitrile hydratases seem to be more abundant in nature as compared to the respective iron-dependent counterparts, as judged by their much higher prevalence in public databases. Herein we extended the chemists’ toolbox for enzymatic amide formation from nitrile precursors and associated experimentally confirmed function to more than a dozen putative NHases. Functional expression of NHases was done in the presence of their native accessory proteins under the control of the tac promoter. Gentle expression at low temperature and supply of active site metal was important. Nevertheless, expression levels strongly varied: whereas some NHases gave high content of soluble protein, the majority of the investigated proteins gave medium levels and only few gave levels close to the detection limit. Of the four new Fe-dependent NHases, three showed promising activity for the four investigated substrates. Fe-dependent NHases were active in the pH range between pH 6.0–8.0. By contrast, Co-dependent NHases were tolerant to a much broader pH range up to pH 9.5. We did not observe a strict substrate preference indicating broad applicability of both Fe- and Co-dependent nitrile hydratases.

## 3. Materials and Methods

### 3.1. General

Tris was purchased from Carl Roth (Karlsruhe, Germany), isopropyl β-d-1-thiogalactopyranoside (IPTG) from Serva (Heidelberg, Germany), methacrylonitrile from Fluka (Buchs, Switzerland) and CoCl_2_ and FeSO_4_*7H_2_O from Merck. HPLCMS grade acetonitrile was purchased from J.T.Baker/Avantor Performance Materials (Deventer, The Netherlands). All other chemicals were obtained from Sigma–Aldrich (St. Luis, MO, USA) and used without further purification.

*E. coli* cells were cultivated in an RS 306 shaker (Infors, Bottmingen, Switzerland), a Multitron shaker (Infors AG Bottmingen, Switzerland) and a Certomat BS-1, and the cells were harvested with an Avanti J-20 XP centrifuge (Beckman Coulter, Brea, CA, USA). Cell pellets were disrupted by a 102C converter with a Sonifier 250 (Branson, Danbury, CT, USA), and the cell-free extract was obtained by centrifugation in an Avanti J-20 XP centrifuge (Beckman Coulter). Reactions were performed on a Thermomixer comfort (Eppendorf, Hamburg, Germany). HPLC/MS analysis was carried out on an Agilent Technologies (Santa Clara, CA, USA) 1200 Series equipped with G1379B degasser, G1312B binary pump SL, G1367C HiP-ALS SL autosampler, a G1314C VWD SL UV detector, G1316B TCC SL column oven and a G1956B MSD. A positive electrospray ionization mode was used as ionization method.

### 3.2. Sequence Identification and Cloning

*Bradyrhizobium japonicum* NHase (*Bj*NHase; BAC49763.1 and WP_028174056.1) was used as a template to search for Co-type NHases whereas for Fe-containing NHases *Rhodococcus erytrhopolis* NHase (*Re*NHase) served as the template for a blastp search against the Gen-Bank non-redundant protein sequences. Sequences were selected based on the following criteria (partly from literature): Presence of activator gene, stability, and probability of efficient expression in *E. coli*. The following sequences were chosen: *Pseudomonas marginalis* NHase *(Pm*NHase; WP_074846646.1 and WP_074846644.1), *Acinetobacter baylyi* NHase (*Ac*NHase; ENV54396.1 and ENV54397.1), *Gordonia hydrophobica* NHase (*Gh*NHase; WP_066163464.1 and WP_066163466.1), *Tardiphaga robiniae* NHase (*Tr*NHase; KZD20487.1 and KZD20486.1), *Bacillus sp.* NHase (*Br*NHase: AAO23015.1 and AAO23014.1), *Nitriliruptor alkaliphilus* NHase (*Na*NHase: WP_052668589.1 and WP_052668588.1), *Pseudonocardia thermophila* NHase (*Pt*NHase; WP_073455624.1 and WP_073455623.1), *Paenibacillus chondroitinus* NHase (*Pc*NHase: WP_047675415.1 and WP_047675418.1), *Comamonas testosteroni* NHase (*Ct*NHase; AAU87542.1 and AAU87543.1), *Klebsiella oxytoca* NHase (*Ko*NHase; OSY94202.1 and WP_0109240358.1), *Roseobacter sp*. NHase (*Rm*NHase: OIQ35619.1 and OIQ35618.1), *Ralstonia solanacearum* NHase (*Rs*NHase; AMP38431.1 and AMP38430.1), *Afipia broomeae* NHase (*Ab*NHase; EKS37369.1 and EKS37368.1), *Variovorax sp.* NHase (*Vv*NHase; WP_042672800.1 and WP_007829432.1), *Microvirga lotononidis* NHase (*Ml*NHase; EIM25394.1 and EIM25395.1), *Aurantimonas manganoxydans* NHase (*Am*NHase; WP_009208459.1 and WP_009208458.1), *Rhizobium leguminosarum* NHase (*Rl*NHase; EJC80161.1 and EJC80160.1), and *Caballeronia jiangsuensis* NHase (*Cj*NHase; WP_035501544.1 and WP_035501545.1).

NHase operons comprise of three open reading frames (nhA, nhB and activator) which are often overlapping. Most genes were ordered at IDT (Coralville, IA, USA), GenScript (Piscataway, NJ, USA) and ThermoFisher Scientific (Waltham, MA, USA) beginning with the start codon ATG of the first subunit and ending with one of the two stop codons TGA/TAA of the activator with overhangs for Gibson assembly with linearized pMS470 vector. The genes encoding *Cj*NHase, *Gh*NHase, and *Pt*NHase were purchased in codon optimized form. *E. coli* Top10F’ were transformed, colonies selected on LB-Amp and colony PCR (polymerase chain reaction) and/or control cuts of isolated plasmids were used to identify positive clones. After sequencing, *E. coli* BL21 (DE3) Gold was transfected with the respective plasmids.

### 3.3. Protein Expression

The strains were cultured at 37 °C in LB-Amp and induction was performed at an optical density at 600 nm (OD_600_) of 0.8–1.0 with 0.1 mM IPTG and 1 mM or 2.5 mM FeSO_4*_7H_2_O, 0.1 mM or 1 mM CoCl_2_ at 20 °C. Pellets were harvested by centrifugation after ~24 h and analyzed after cell disruption with BugBuster reagent by SDS-PAGE.

Cell free extracts (CFE) for biotransformation reactions were prepared as follows: Cultivation was performed as described above, typically in a total volume of 400 mL LB medium, using 1 L baffled Erlenmeyer flasks. Cells were harvested by centrifugation at 5,000× *g* and 4 °C for 15 min. The supernatant was discarded and 1.2–3.0 g of pellets were resuspended in 25 mL of 50/40 mM Tris-butyrate buffer, pH 7.2, and lysed on ice by sonication for 6 min at 70–80% duty cycle and 7–8 output control. Cell-free extracts were obtained after centrifugation for 1 h at 48,250× *g* (1 mM FeSO_4*_7H_2_O and 0.1 mM CoCl_2_ samples) or 20,000× *g* (2.5 mM FeSO_4*_7H_2_O and 1 mM CoCl_2_ samples). The temperature was 4 °C and samples were filtered through 0.45 µm syringe filters. Protein concentration was determined using the Pierce™ BCA Protein Assay Kit (ThermoFisher).

### 3.4. Spectrophotometric MAN Assay

Physicochemical characteristics of NHases were determined by monitoring the hydrolysis of methacrylonitrile (MAN, **1a**). Therefore, 10 µL NHase-CFE (diluted in Tris/butyrate buffer 50/40 mM pH 7.2) were mixed with 100 µL of 125 mM MAN in Tris/butyrate buffer 50/40 mM (pH 7.2) in 96-well UV star plates. The formation of methacrylamide (MAD, **1b**) was monitored at 224 nm on a Synergy Mx Plate-reader (BioTek Instruments, Winooski, USA) at 25 °C for 5 min. The Units of the sample can be calculated with Equation (1):

(1) $$\frac{U}{ml}=\frac{Velocity\;\left(min^{-1}\right)\;x\;0.11\;mL\;x\;dilution}{2.551\;mMcm^{-1}\;x\;0.1\;mL\;x\;pathlength\;\left(cm\right)}$$

Appropriate blank reactions were carried out in parallel and each reaction was carried out at least in triplicate.

For the determination of the pH optimum, the standard assay as described above was used with the following buffers: 100 mM citrate-phosphate buffer pH 5–6, 100 mM sodium phosphate buffer pH 7–8, 100 mM Tris-HCl buffer pH 8.5, 100 mM carbonate buffer pH 9–10.

### 3.5. Synthesis

Racemic 3-amino-3-(p-toluoyl)propanenitrile (**3a**) was prepared as previously published and all analytical data corresponded [20]. The corresponding amide **3b** was prepared as follows: A solution of *rac*-3-amino-3-(p-toluoyl)propanenitrile (**3a**, 30 mg, 0.187 mmol) in MeOH (1 mL) was added to a suspension of *Rhodococcus rhodochrous* ATCC BAA-870 [25] (30 mg wet weight cells) in 50 mM Tris buffer (pH 7.6, 4 mL). The mixture was stirred vigorously on a magnetic stirrer unit for 24 h. Ethyl acetate (5 mL) was added and the mixture was vortexed well followed by centrifugation at 10,000× *g* for 10 min. The top organic layer was dried over MgSO_4_, filtered and dried in vacuo to yield almost pure amide product. After trituration in hexane and filtration of the product, the racemic amide was obtained as a white solid (26 mg, 78%). All analytical data corresponded to that previously published [20].

### 3.6. Chromatographic Assay

The typical assay to determine directly the reaction products was carried out as follows: 100 µL NHase-CFE (16–96 µg/mL for **2a** and **3a**, 32-192 µg/mL for **4a**) were mixed with 100 µL substrate solution (10 mM of **2a**, **3a**, or **4a** in reaction buffer) and incubated for 5 min at 25 °C and 350–450 rpm in Eppendorf thermomixers. The reactions were stopped by the addition of 400 µL MeOH/HCOOH (19:1). After centrifugation of precipitated protein, the supernatants were analyzed by HPLC. The nitriles were separated from amides by a Phenomenex (Torrance, USA) Kinetex 2.6 µm Biphenyl 100Å column (150 × 2.1 mm, 2.6 µm) using 5 mM ammonium acetate solution and acetonitrile as the mobile phase, at a flow rate of 0.26 mL/min for 15 min. A gradient with increasing acetonitrile levels was used: 25–55% (5 min), 55–70% (2 min, 20 s), 70–90% (30 s), 90% (1 min, 10s), 90–25% (1 s), 25% (5 min, 59 s). The compounds were detected at 210 nm (diode array detector).

## Figures and Tables

**Table 1:** Screening cell free extracts for nitrile hydratase activity towards hydrolysis of methacrylonitrile.

NHase	Estimated NHase Content in CFE [mg/mL]	Activity[µmol min^−1^ mg^−1^]^1^	Specific Activity [µmol min^−1^ mg^−1^]^2^
Fe-type *Ac*NHase	<0.1	3	0
Fe-type *Gh*NHase	3.4	18	55
Fe-type *Pk*NHase	2.9	182	615
Fe-type *Pm*NHase	1.9	52	313
Fe-type *Re*NHase	2.9	248	605
Co-type *Ab*NHase	1.6	14	53
Co-type *Am*NHase	0.8	3	37
Co-type *Bj*NHase	0.1	2	14
Co-type *Br*NHase	0.1	4	20
Co-type *Cj*NHase	2.9	23	88
Co-type *Ct*NHase	3.4	118	336
Co-type *Ko*NHase	2.9	165	613
Co-type *Ml*NHase	3.9	30	98
Co-type *Na*NHase	1.0	62	505
Co-type *Pc*NHase	1.7	1	5
Co-type *Pt*NHase	1.2	41	243
Co-type *Rl*NHase ^3^	0.4	0	0
Co-type *Rm*NHase	4.2	45	138
Co-type *Rs*NHase	2.0	0	0
Co-type *Tr*NHase	1.7	1	3
Co-type *Vv*NHase	1.5	8	54

^1^ Activity relates to total protein content of the cell free extract (CFE). ^2^ Specific activity calculated on basis of estimated NHase content in CFE. ^3^ Addition of 1 mM of CoCl_2_ at induction.

## Supplementary Materials

The following are available online at https://www.mdpi.com/1420-3049/25/11/2521/s1, Figure S1–S5: SDS-PAGE of NHases.
