# Supplementary material for: Functional Expression and Characterization of a Panel of Cobalt and Iron-Dependent Nitrile Hydratases
Source: Molecules. 2020 May 28;25(11):2521. doi: 10.3390/molecules25112521 (PMC7321127; doi:10.3390/molecules25112521)
Supplement: Supplementary file 1 [file molecules-25-02521-s001.pdf]

Functional expression and characterization of a panel of cobalt and iron dependent nitrile hydratases

Birgit Grill, Maximilian Glänzer, Helmut Schwab, Kerstin Steiner, Daniel Pienaar, Dean Brady, Kai Donsbach, and Margit Winkler

Supplementary Information

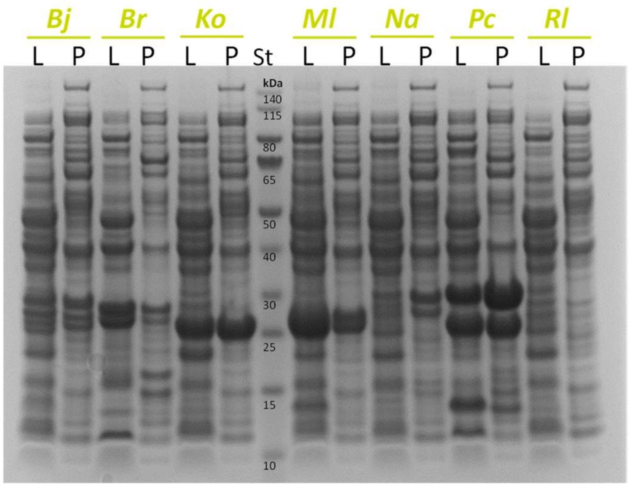

Figure S1. SDS-PA gel of expression of Co-type NHases. Cell-free lysate (L); insoluble pellet fraction (P). Expected sizes of NHase subunits (in kDa): *Bj* α 26, β 25; *Br* α 25, β 26.5; *Ko* α 22.5, β 24.5; *Ml* α 23, β 24; *Na* α 24, β 26; *Pc* α 25, β 27; *Rl* α 29, β 24. St: PageRuler Prestained Protein Ladder.

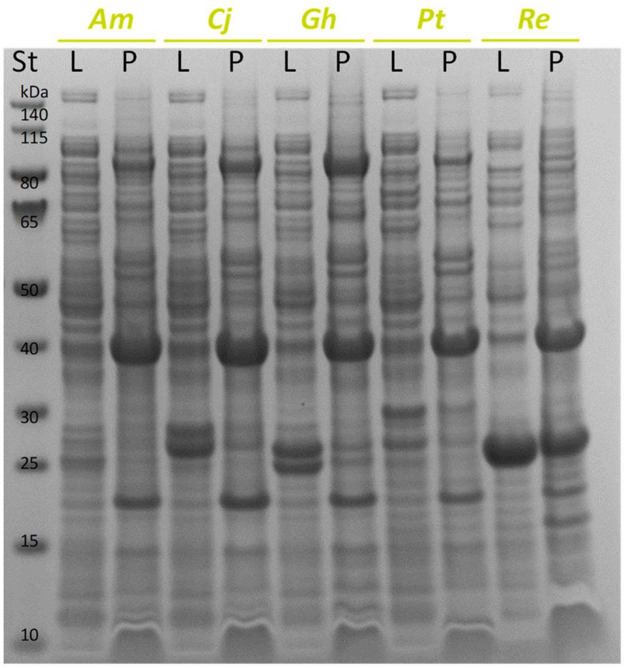

Figure S2. SDS-PA gel of expression of NHases. Cell-free lysate (L); insoluble pellet fraction (P) Expected sized of NHases subunits (in kDa): *Am* α + β 24; *Cj* α + β 24; *Gh* α 23, β 24; *Pt* α 23, β 26.5; *Re* α 23, β 23.5. St: PageRuler Prestained Protein Ladder.

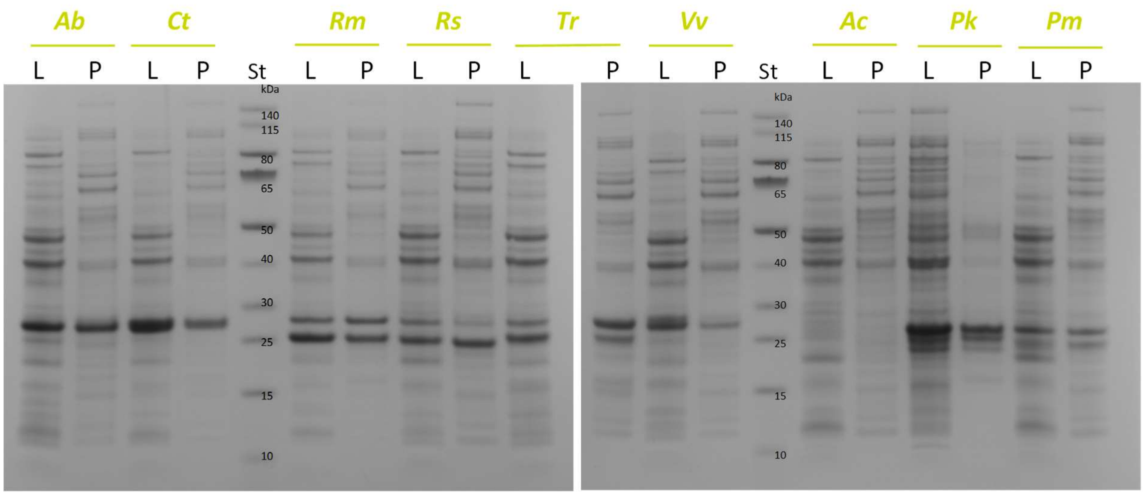

Figure S3. SDS-PA gel of expression of NHases. Cell-free lysate (L); insoluble pellet fraction (P) Expected sizes of NHase subunits (in kDa): *Ab* α 25, β 24; *Ct* α 23, β 24; *Rm* α 23, β 25; *Rs* α 23, β 24; *Tr* α 23, β 27; *Vv* α 24, β 24; *Ac* α 22, β 24; *Pk* α 22, β 24; *Pm* α 22, β 24;. St: PageRuler Prestained Protein Ladder.

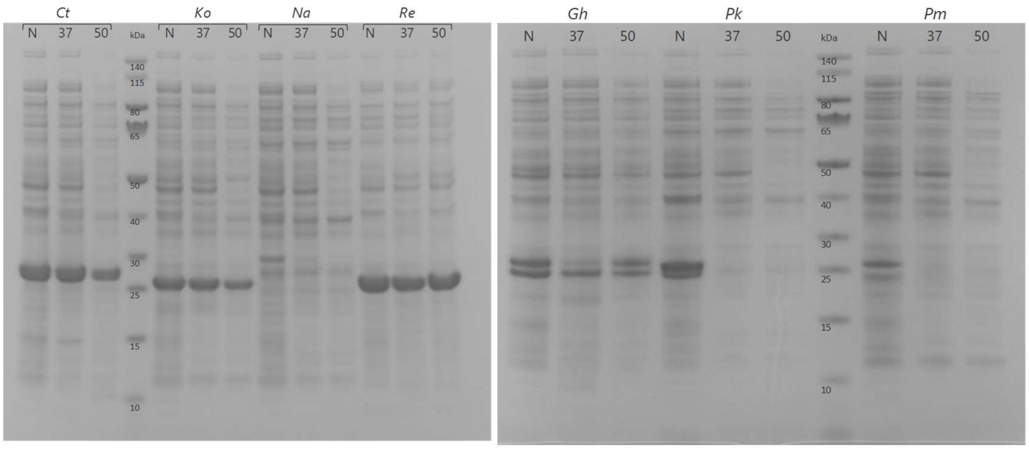

Figure S4. SDS-PA gel of NHase-CFE incubated at pH 7.2 at 37°C or 50°C overnight compared to non-incubated CFE (N). Expected sizes of NHase subunits (in kDa): *Ct* α 23, β 24; *Ko* α 22.5, β 24.5; *Na* α 24, β 26; *Re* α 23, β 23.5; *Gh* α 23, β 24; *Pk* α 22, β 24; *Pm* α 22, β 24;. St: PageRuler Prestained Protein Ladder.

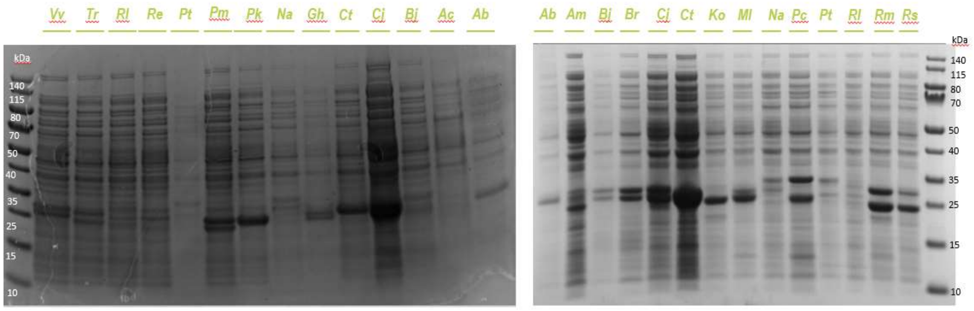

Figure S5. SDS-PA gel of NHase-CFE cultivated in the presence of 1 mM $CoCl_2$ or 2.5 mM $FeSO_4$, respectively. Expected sizes of NHase subunits see Figures above. St: PageRuler Prestained Protein Ladder.
